# An Improved YOLOv8 Model for Pavement Distress Detection Under Low-Computing-Power Conditions

**DOI:** 10.3390/s26113373

**Published:** 2026-05-26

**Authors:** Yi Tang, Ziyi Yang, Zhoucong Xu, You Zhou, Hui Wang

**Affiliations:** 1 China Merchants Expressway Network & Technology Holdings Co., Ltd., Beijing 100029, China; tangyi@cmhk.com; 2 State Key Laboratory of Safety and Resilience of Civil Engineering in Mountain Area, School of Civil Engineering, Chongqing University, Chongqing 400045, China; zy_yanghhh@163.com; 3 China Merchants Chongqing Communications Technology Research & Design Institute Co., Ltd., Chongqing 400067, China; xuzhoucong@cmhk.com (Z.X.); zhouyou3@cmhk.com (Y.Z.)

**Keywords:** pavement distress detection, routine survey, multi-objective detection, real-time, YOLOv8

## Abstract

**Highlights:**

**What are the main findings?**

- The proposed model shows advantages over 10 SOTA models in pavement detection.
- Integrates LSKA, DIoU loss, and Soft-NMS into YOLOv8n, achieving 78.3% mAP@0.5.

**What are the implications of the main findings?**

- Pothole detection AP↑22.1%, strip patch detection AP↑17.7%.
- Maintains 160 FPS (GPU) and 68 FPS (low-cost CPU laptop).

**Abstract:**

Automated pavement distress detection (PDD) is critical for the structural health monitoring (SHM) of transportation infrastructure, yet existing methods struggle with real-time multi-target detection under resource constraints. In this paper, YOLOv8-PDD was constructed based on YOLOv8 by introducing the large separable kernel attention (LSKA) mechanism module into the Spatial Pyramid Pooling—Fast (SPPF) module, replacing Complete-IoU (CIoU) loss with Distance-IoU (DIOU) loss as the loss function, and adopting Soft-Non-Maximum Suppression (NMS) to replace the original NMS algorithm. The proposed YOLOv8-PDD achieved 78.3% mean average precision with intersection over union above 0.5 (mAP@0.5 +8.1%) with a minimal complexity increase of +0.2 GFLOPs compared to the baseline YOLOv8n model. While incurring a negligible increase in latency (+0.09 ms), YOLOv8-PDD significantly outperforms YOLOv8n in detection accuracy (mAP@0.5 +8.1%), offering a superior accuracy–efficiency trade-off for real-time applications. YOLOv8-PDD performed well in detecting all categories, with AP values above 75% except for transverse crack and strip patch. Significant improvements in pothole detection AP@0.5 (+22.1%) and strip patch detection AP@0.5 (+17.7%) indicate superior small target and complex background adaptability. Our model achieved a detection efficiency of 68 frames per second (FPS) on consumer-grade CPUs (OpenVINO-optimized), outperforming 10 models (e.g., YOLOv5n and RTDETR-l) in accuracy–speed balance.

## 1. Introduction

### 1.1. Background

The advancement of deep learning target detection technology has led to the gradual evolution of pavement distress detection (PDD) towards being multi-objective, highly accurate, and highly efficient [1,2,3]. The development of lightweight detection models for smartphones, unmanned aerial vehicles (UAVs), and car recorders provides the possibility of embedded detection scenarios, which are expected to realize the real-time output of PDD results [4,5,6,7]. However, the real-time detection capabilities of established lightweight detection algorithms are mostly based on efficiency tests in GPU mode, and the embedded scenario applications are less frequently addressed, especially when involved in a multi-target object detection task.

To reduce the requirements for UAV connectivity and bandwidth, some scholars have considered computational efficiency in embedded systems when designing detection networks, which have been discussed in references [8,9,10,11,12]. However, these studies on embedded scenarios frequently focused on a single distress type—crack. Moreover, the majority of the efficiency metrics were not tested on CPU devices or embedded systems [13,14]. Notably, the concept of efficiency is absent from some studies on the UAV-based detection of multiple distress targets. A variety of real-time detection scenarios involving pavement distress are available for in-vehicle platforms, which affords in-vehicle equipment a greater potential for high-frequency detection of pavement distress. Several multi-category distress detection efficiency analyses based on low-cost vehicle-mounted cameras have been conducted [4,5,6]. However, the application of these models to specific embedded platforms remains to be investigated.

### 1.2. Related Works

Convolutional neural network (CNN)-based target detection methods can be broadly classified into two categories: two-stage models and one-stage models [15]. The two-stage model initially generates a substantial number of potential frames that may contain the target through a candidate frame generator. Subsequently, these frames undergo further classification and regression to ascertain the precise target location and bounding box. Notable examples of representative algorithms include RCNN (Region-CNN) [16] and Faster R-CNN [17]. Liu et al. [18] integrated Faster R-CNN with image segmentation techniques to detect and segment pavement distress (cracks, potholes, and patches). This approach reduces computational costs while offering a new avenue for real-time PDD tasks. In contrast to the two-stage model, the single-stage model accomplishes all the tasks of target detection in a single forward propagation. This is achieved by combining the classification and regression tasks into a single network for end-to-end training. Notable examples include You Only Look Once (YOLO) [19] and Single Shot MultiBox Detector (SSD) [20]. Unlike traditional CNNs, Transformer employs the self-attention mechanism to process image data. The fundamental concept of the Transformer-based detector, DETR [21], is to transform the target detection problem into an ensemble prediction problem. Dai et al. [22] proposed a pre-training task called random query patch detection for unsupervised pre-training DETR, which markedly enhanced the performance of DETR with a faster convergence rate and higher average accuracy in object detection, one-time detection, and panorama segmentation.

Sun et al. [23] enhanced the SSD algorithm by incorporating an anti-convolutional feature module and an attention mechanism, thereby achieving superior performance in the task of detecting a small-targeted pedestrian in a large-scale input image relative to the SSD performance. Lin et al. put forth an efficient Transformer-based detection model for the nondestructive discrimination of pavement anomalies [24]. Du et al. [25] assembled a comprehensive dataset of pavement distress and employed YOLO to ascertain the distress’s location and category within the image. This approach yielded a combined detection accuracy of 73.64% and a processing speed of 0.0347 s/image, which is nine times faster than Faster R-CNN and only 70% of SSD.

The development of UAV technology has brought a new development path for the timely detection of road damage, with the significant advantage of wider inspection ranges [8,26]. Nevertheless, with the combination of UAVs and deep learning, and its application to PDD tasks, there is still some distance from engineering applications due to the limitations of the hardware resources of the carrying platform [2]. In the context of UAV applications, Zhang et al. [13] and Zhu et al. [7] developed YOLOv3-based models, achieving mean average precision (mAP) values of 0.6875 and 0.566, respectively. Alonso et al. [9] designed an EfficientNet-FPN network optimized with TensorRT for use with the embedded system Nvidia Jetson AGX Xavier. Subsequently, the latency associated with crack detection in images of varying resolutions of the airfield roadway surface was evaluated, yielding results between 53 and 111 milliseconds.

In-vehicle video technology represents a more cost-effective approach to pavement distress surveys. Lei et al. [27] and Ren et al. [4] achieved high mAP values for multi-target detection based on Baidu API data using YOLOv3 and YOLOv5 models, respectively. Lee proposed the use of adaptive frame control (AFC) as a solution to the real-time delay issue inherent to YOLO [28]. A YOLOv5-based model demonstrated superior efficiency in detecting four types of distress when compared to Faster R-CNN in a lightweight model study based on image data collected by a front-view low-cost camera [5]. A similar data collection method revealed that YOLOv5s-M exhibited superior efficiency and accuracy compared to YOLOv7 in the detection of seven types of distress [4]. The use of image data collected by front-view car recorders to detect 12 distress targets demonstrated that YOLOv7-tiny outperformed the algorithmic frameworks, including YOLOv4-tiny and YOLOv5-tiny, in terms of efficiency metrics while maintaining acceptable accuracy. This resulted in the proposal of a more precise and efficient model, namely YOLOv7-RDD [6].

Despite the numerous iterations of YOLO that have been conducted, with incremental enhancements in precision, the influence of real-time streaming protocol inputs on resource-constrained platforms may potentially give rise to a cumulative delay issue, which has not been adequately addressed. The implementation of distress detection programs with processing speeds that are lower than the frame rate transmitted by the camera will inevitably result in significant challenges with regard to real-time processing. Despite the availability of multiple efficiency metrics, the specific embedded platform requirements for the application remain unclear. Unlike existing studies focusing on high-end embedded systems (e.g., Jetson AGX), this work specifically addresses the challenge of deploying pavement inspection systems on low-cost, readily available consumer tablets.

## 2. Methods

### 2.1. YOLOv8

The overall structure of YOLOv8 comprises four distinct components: the input, the backbone network (Backbone), the neck network (Neck), and the head network (Head). The input side is responsible for preprocessing the input image, which includes mosaic data enhancement and adaptive image scaling. The model employs convolutional operations to downsample the image and extract features. Furthermore, a novel C2f module has been incorporated into YOLOv8, drawing inspiration from the E-ELAN (extend efficient layer aggregation network) structure present in YOLOv7 [29] to enhance the model gradient flow and elevate the detection outcomes by interconnecting branches across layers. The backbone network comprises five convolutional modules, four C2F modules, and one SPPF (Spatial Pyramid Pooling—Fast) module. Each convolutional module contains a 2D convolution, batch normalization, and a SiLU activation function. The SPPF module, situated after the backbone network, employs three maximal pooling layers to process multi-scale features, thereby facilitating the extraction of more nuanced and representative features. The primary function of the neck network is to integrate the diverse scale feature maps generated by the backbone network. Its fundamental structure encompasses FPNs (feature pyramid networks) [30] and a PAN (path aggregation network) [31]. A FPN is employed to construct a feature pyramid by extracting features from varying scales of the image, whereas the PAN is utilized to aggregate these features across disparate layers of the network. Ultimately, the neck network transmits the feature data to the head network. In YOLOv8, the head network employs a decoupled detection head to compute the regression and category losses through two parallel convolutional branches. In practical pavement distress detection work, it is necessary to balance detection accuracy and speed. YOLOv8n, as the smallest variant in the YOLOv8 series, has been chosen as the baseline model in this paper.

### 2.2. Improved YOLOv8

In the actual pavement distress detection process, the complexity of the detection background will have a certain impact on the distress detection results, especially when detecting certain small targets (such as potholes and manhole covers) appearing in the detection screen. The detection effect of YOLOv8n is not ideal, and it is easy to miss the targets. In addition, the overlap rate of the same type of distress targets at the same location is high, and the use of the original non-maximum suppression (NMS) algorithm may also lead to missed detection.

**Figure 1:**
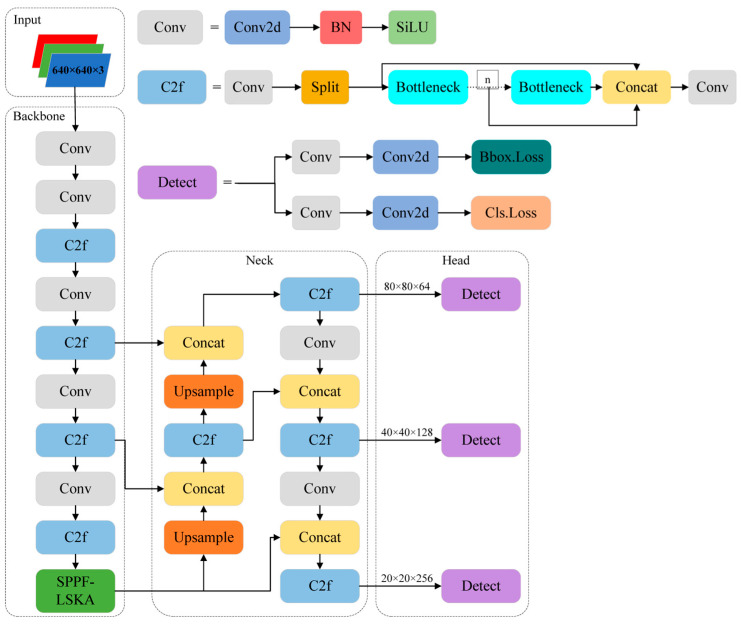
The structure of the improved YOLOv8n model.

To solve the above problems, an improved YOLOv8n model is proposed, and Figure 1 shows the structure of the model. First, the large separable kernel attention (LSKA) [32] mechanism is introduced into the SPPF module in the YOLOv8 backbone network, so that the network ignores the interference of irrelevant background information and notices more effective distress feature information. Secondly, Distance-IoU (DIOU) loss [33] is used to replace Complete-IoU (CIoU) loss in the original model to overcome the problem of lower detection accuracy when there are large-scale and location changes in distress targets. Finally, the Soft-NMS algorithm [34] is chosen to replace the NMS algorithm in the original network.

The three selected modules target distinct aspects of pavement distress detection. LSKA addresses background clutter by enlarging the receptive field without heavy computation, DIoU improves regression convergence for small or distant distress, and Soft-NMS mitigates missed detections in dense distress scenarios.

### 2.3. LSKA Mechanism

**Figure 2:**
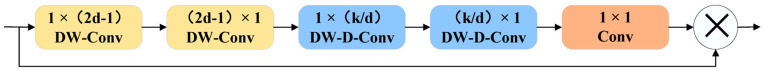
The structure of LSKA.

**Figure 3:**
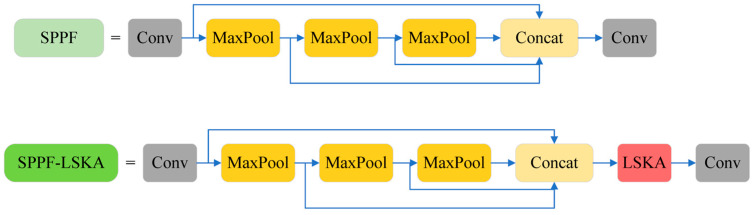
The structures of SPFF and SPPF-LSKA modules.

Pavement distress often exhibit elongated structures (cracks) or small, scattered patterns (potholes). LSKA’s large separable kernel captures long-range dependencies (e.g., a continuous crack), while the decomposition into 1D kernels keeps computational cost low. Compared to standard attention, LSKA is particularly suitable for low-power devices because it adds minimal FLOPs (+0.2 G) while suppressing irrelevant background textures such as shadows or road markings. LSKA is an innovative large separable kernel attention module that captures long-range dependencies and adaptations by decomposing large kernel convolution operations, thereby reducing computational complexity and memory requirements. LSKA decomposes a 2D convolution kernel of a deep convolutional layer into cascaded horizontal and vertical 1D convolution kernels. This is achieved by first decomposing a convolution kernel of size *K* × *K* into multiple parts, including the (2*d* − 1) × (2*d* − 1) depth convolution kernel, *K*/*d* × *K*/*d* depth dilation convolution kernel, and 1 × 1 convolution kernel. Secondly, these 2D depth convolution kernels with depth dilation convolution kernels are decomposed into smaller 1D horizontal and vertical convolution kernels. Finally, the decomposed convolution kernels are cascaded sequentially. Figure 2 shows the structure of LSKA, where ⊗ represents the Hadamard product, *k* represents the maximum sensory field, and *d* represents the dilation rate. The structure of the original SPPF module and the improved SPPF-LSKA module after the introduction of LSKA is shown in Figure 3.

The effectiveness of LSKA for small-scale and irregular pavement distress stems from its contextual contrast. Standard kernels often confuse small potholes or thin cracks with pavement textures. By utilizing a large receptive field, LSKA provides the model with a broader environmental context, allowing it to differentiate between stochastic noise and structured distress patterns. A theoretical comparison of LSKA with other widely used attention modules is in Table 1.

The comparison presented in Table 1 is grounded in the architectural evolution of attention mechanisms. SE and CBAM represent localized or channel-wise approaches that struggle to preserve the long-range structural continuity of pavement cracks. While Transformers offer global receptive fields, their quadratic computational complexity and the loss of local inductive bias (due to patch embedding) make them less suitable for high-resolution, real-time pavement inspection on edge devices.

LSKA is uniquely advantageous in this context as it mimics the global self-attention mechanism via large-kernel convolutions. By retaining the structured nature of convolutions while expanding the effective receptive field, LSKA strikes an optimal balance that may be powerful enough to model the entire path of a crack yet efficient enough to be integrated into the lightweight YOLOv8n backbone. This theoretical alignment with the geometric properties of front-view-based pavement distress justifies its selection over other generic attention modules.

As shown in Figure 3, the feature map X is first obtained by one convolutional layer, then fed into three maximal pooling (MaxPool) layers to obtain three different scales of feature maps, which are cascaded as inputs to LSKA. After the LSKA process, new feature maps are obtained and finally processed by one convolutional layer to obtain the final output.

### 2.4. Loss Function

The original YOLOv8 model used distribution focal loss (DFL) [20] and CIoU loss as regression loss functions. For a pavement distress detection dataset, the distress targets often have large-scale and positional variations, so choosing a loss function that pays more attention to the distance between the centroids of the prediction frame and the real frame helps the model to better capture the positional variations of the targets, thus improving the accuracy of detection. CIoU includes an aspect ratio penalty term that may become unstable when detecting distress with high shape variability (e.g., cracks of uneven width). DIoU simplifies the penalty to centroid distance, which is more robust for small, non-rigid targets such as potholes and strip patches. Our experiments confirm that DIoU leads to faster convergence and higher AP for these categories. The Euclidean distance between each detection box is calculated in DIoU loss, which takes the distance between the target box and the centroid of the prediction box, the overlap rate, and scale inconsistency into account, making the target box regression more stable. DIoU loss can be expressed as in (1).

(1) $$L_{DIoU}=1-IoU+\frac{{\left(x-x^{gt}\right)}^{2}+{\left(y-y^{gt}\right)}^{2}}{c^{2}}$$

where (x, y) and ($x^{gt}$, $y^{gt}$) represent the coordinates of the centers of the predicted and ground-truth boxes, respectively, *c* represents the diagonal length of the smallest closed region that can contain both the prediction frame and the real frame, and IoU is expressed in (2).

(2) $$IoU=\frac{\left|B\cap B^{gt}\right|}{\left|B\cup B^{gt}\right|}$$

where *B* represents the prediction frame and $B^{gt}$ represents the true frame.

### 2.5. Soft-Non-Maximal Suppression (Soft-NMS)

The NMS algorithm is an important part of the target detection model, which will rank the target confidence of all prediction frames and select the prediction frame with the highest confidence, removing frames that overlap with the prediction frames within a predetermined threshold range. However, the NMS algorithm used in YOLOv8 has a problem in that, when a target is highly overlapped with a prediction frame, it may be removed directly, which is the main reason for the low accuracy of overlapping target detection. In the actual process of pavement distress detection, the same type of distress is often concentrated in one place (e.g., cracks and strip repairs), and the target overlap rate is high; therefore, the traditional NMS algorithm is not applicable. To solve this problem, we chose the Soft-NMS algorithm [17] to replace the NMS algorithm in the original network.

The Soft-NMS algorithm introduces a penalty function for prediction frames that are above a threshold, reducing the score of the detected frames instead of directly setting them to zero. That is, when the overlap of the candidate frames exceeds the threshold, instead of rejecting the candidate frames directly, the decay function reduces the scores of the overlapping frames to retain some frames that will be suppressed in the future. *B* represents the set of prediction boxes, *b_i_* represents a certain prediction box in the set, *S_i_* is the value of *b_i_* confidence, *M* is set to be the prediction box with the largest confidence value in *B*, *IoU* (*M*, *b_i_*) represents the intersection over union between *b_i_* and *M*, and *N_t_* represents the IoU threshold that needs to be suppressed. There are two ways of changing the confidence level in the soft-NMS calculation method, including linear weighting and Gaussian weighting. The linear weighting is shown in (3).

(3) $$S_{i}=\{\begin{array}{c} S_{i},\;IoU\left(M,b_{i}\right)<N_{t}\\ S_{i}\left(1-IoU\left(M,b_{i}\right)\right),\;IoU\left(M,b_{i}\right)\geq N_{t} \end{array}$$

The higher the overlap between the predicted border *b_i_* and the selected border *M*, the more obvious the suppression effect of soft-NMS and the smaller the updated confidence *S_i_*. Conversely, the inhibition is weaker, and the updated confidence *S_i_* is larger.

The Gaussian weighting-based approach is expressed in (4).

(4) $$S_{i}=S_{i}e^{-\frac{IoU\left(M,b_{i}\right)}{\sigma }},\;\forall b_{i}\notin D$$

where *σ* is a nonlinear coefficient used to compute and control the Gaussian penalty term, such that the suppression strength of Soft-NMS on edges increases as *M* increases with *IoU*. With this approach, some of the higher-scoring prediction frames are retained and may still be considered correct in subsequent processing, thus alleviating the problem of model under-detection.

## 3. Experiment

### 3.1. Dataset

**Figure 4:**
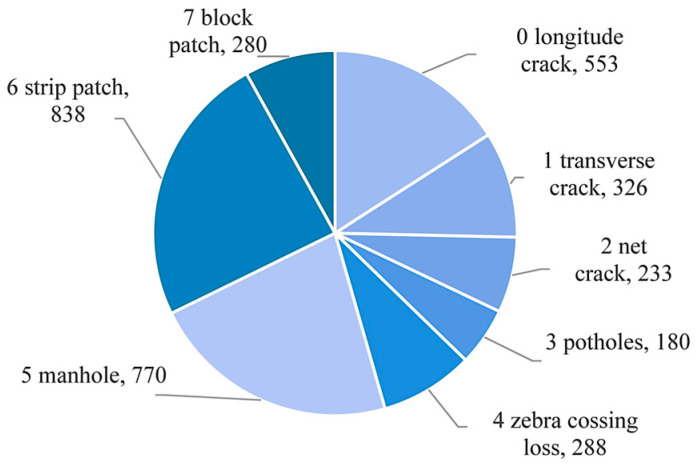
Distribution of the dataset labels.

The constructed base dataset contains 1925 images, 3468 distress labels, and a total of eight categories of distress. Among them, 1228 are from RDD2022 [38], based on which the hand-labeled categories are expanded (zebra crossing loss, manhole cover, strip repair, and block repair are included), and the remaining 697 images were collected in Shanghai and Chongqing. The distribution of each sample category is shown in Figure 4. The training and testing data from Japan in RDD2022 include both urban and suburban areas, accounting for more than half of the dataset, with some data captured on cloudy days or on wet pavement surfaces. The U.S. data constitute about five-sixths of the dataset and were all collected under sunny conditions, with generally dusty roads and light-colored pavement. The remaining data from Brazil have a lower original resolution and exhibit relatively severe pavement distress. The data from Shanghai, China (532 images) feature a consistent camera perspective, are all from urban roads, contain frequent road patches, and have high original resolution; repetitive defects caused by continuous shooting (where the target size changes but remains visible) have been removed. The Chongqing data mainly consist of frames extracted from dashcam footage and suffer from significant distortion.

**Figure 5:**
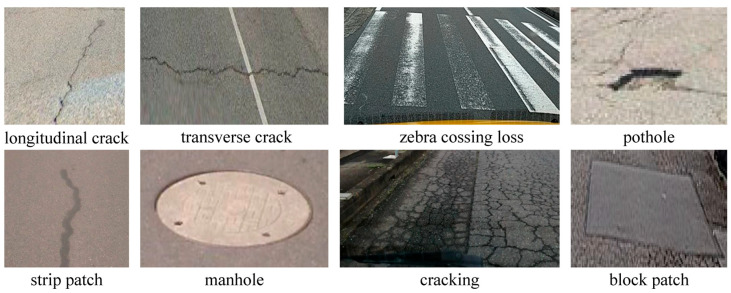
Samples of distress types.

The dataset is divided into training, validation, and test sets in the ratio of 8:1:1. Examples of different types of distress are shown in Figure 5.

To evaluate the improvement and generalization, a public dataset SCVRDD [39] containing 8000 images was used for verification under the same experimental setting for both training and testing.

### 3.2. Experiment Setting and Evaluation Metrics

The experimental environment is based on ubuntu20.04, Python 3.8.10, Pytorch1.11.0+cu11.3 framework, CUDA11.3, and the hardware devices are an 18 vCPU AMD EPYC 9754 128-Core Processor and NVIDIA GeForce RTX 3090. The initial learning rate was set to 0.001, weight decay to 0.0005, epoch to 200, and batch size to 16.

To accurately assess the inference effect in the end-side device, the experiment utilizes the CPU device employed during model testing. The objective is to evaluate the real-time performance of several models in the asynchronous mode of inference calculation experiments on the test set of pavement distress images. The specific experimental program was conducted using the following processes. (1) The inference engine should be initialized based on the OpenVINO framework, and the supported hardware devices should be identified. (2) As the framework does not support direct reading of the PyTorch model, the YOLO model is first transformed into an ONNX format file, then into an intermediate expression model IR file with FP16 precision. This contains the network structure parameter file and network weight parameter file. (3) The deep learning model inference computation performance testing tool, included with the framework, is used to test the inference computation performance of different models. This is done by parsing IR files on specified computing devices, in either synchronous or asynchronous mode.

In this paper, *mAP* and *F*1-*Score* are used to evaluate the model’s accuracy. Frame rate per second (*FPS*), the inference time of the model (*IFT*), and post-processing time (*Latency*) are used to evaluate the detection efficiency. The number of parameters (*Params*), and the number of floating-point operations per second (*FLOPs*) are used to evaluate the model size and complexity. The related evaluation metrics are calculated according to Equations (5)–(9). *TP* denotes a true positive, *FP* denotes a false positive, *TN* denotes a true negative, and *FN* denotes a false negative. *AP* denotes the area enclosed by the coordinates *x* and *y* of the P–R curve, and *N* denotes the number of detection categories.

(5) $$Precision=\frac{TP}{TP+FP}$$

(6) $$Recall=\frac{TP}{TP+FN}$$

(7) $$F1=2*\frac{Precision*Recall}{Precision+Recall}$$

(8) $$AP=\int_{0}^{1}P\left(r\right)dr$$

(9) $$mAP=\frac{1}{N}\sum_{i=1}^{N}AP_{i}$$

where *mAP@0.5* represents the mean value of the average precision (*AP*) for each class of detection target computed when the *IoU* threshold is set to 0.5; *mAP*@0.5:0.95 and the average value of the *mAP* computed for each *IoU* threshold in steps of 0.05 from 0.5 to 0.95.

## 4. Results and Discussion

### 4.1. Evaluation of Testing Results

**Figure 6:**
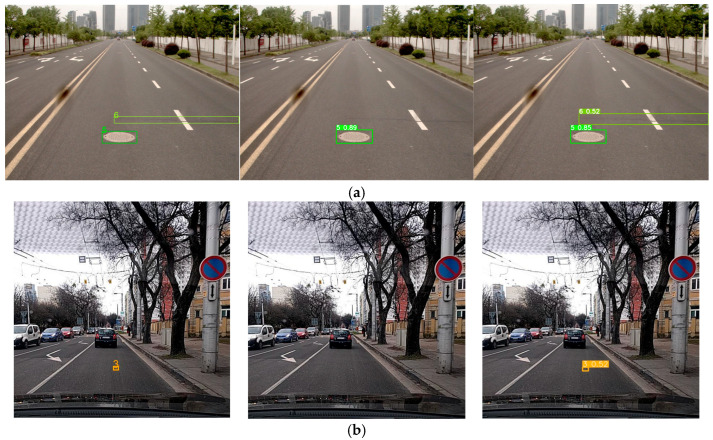

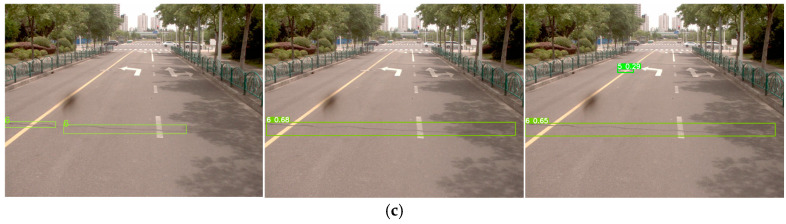
Detection sample comparison. From left to right are labeled images of the results detected by YOLOv8n and our model, respectively. (**a**) Manhole and strip patch, (**b**) pothole, (**c**) strip patches.

The comparison results of YOLOv8n and our improved model based on the test set are shown in Table 2. The *AP* results for different categories based on the test set are shown in Table 3. Examples of the detection results corresponding to the categorized targets are shown in Figure 6.

Table 2 shows that the improved model has increased in *Params, FLOPs,* and *Latency*, but the increase is small, with *Params* increasing by about 0.27 M, model size increasing by about 0.5 M, and *FLOPs* increasing by about 0.2 G. The increased complexity of the model is due to the introduction of the LSKA mechanism. Meanwhile, *FPS* decreased slightly by about 2.3. Inversely, *mAP*@0.5, *mAP*@0.5:0.95, and *F*1 all significantly increased. *mAP*@0.5 increased by about 8.1%, *mAP*@0.5:0.95 increased by about 7.1%, and *F*1 increased by 5%.

As seen in Table 3, the improved model shows a significant improvement in average precision (*AP*) in all categories, except for the category of zebra crossing loss. Particularly noteworthy is that the improvement effect of the improved model is most obvious in the detection of potholes and strip patches, where the *AP* of pothole detection improved from 53.4% to 75.5%, and that of strip patch detection improved from 52.1% to 69.8%. The background of the front-view image is complex, where some targets are easily missed due to their small size, and the LSKA attention mechanism allows the network to ignore the interference of irrelevant background information and notice more effective information about the distress features.

From Figure 6, it can be seen that in the group (a) images, our model accurately localizes the strip patch (class 6) and manhole cover (class 5), while YOLOv8n fails to detect the strip patch (class 6). In the group (b) images, our model localizes the pothole (class 3) of the small target type, while YOLOv8n missed a target. In the group (c) images, both the original YOLOv8n model and our model recognized the strip patch (class 6), and our model also recognized an unlabeled manhole cover (class 5).

The category test results based on the SCVRDD dataset are illustrated in Table 4.

As shown in Table 4, while the mean AP50 increased by 0.8%, the mean AP50–95 showed a more significant gain of 1.1%. This suggests that our integration of the DIoU loss function and Soft-NMS effectively refines the bounding box regression, leading to higher localization accuracy, which is crucial for infrastructure maintenance. The most notable improvement is observed in alligator cracks (+3.7% in AP50). This empirically validates the theoretical advantage of our LSKA module, which captures long-range spatial dependencies and structural continuity better than the standard kernels in the baseline model. In the case of “Potholes”, we observed a slight decrease in mAP50 but a substantial 3.5% increase in mAP50–95. This indicates that our model has become more precise in its predictions, prioritizing high-IoU matches over potential false positives, which is a desirable characteristic for reliable automated inspection. The slight fluctuations in “Longitudinal Patch” detection (a category characterized by large, uniform areas) reflect the model’s specialized adaptation to fine-grained defect detection, a known trade-off in architectural optimizations focusing on small-target feature enhancement.

### 4.2. Ablation Test Analysis

Ablation experiments were conducted on the improved YOLOv8n model, including the introduction of LSKA into the SPPF module, the replacement of the loss function with DIoU, and the replacement of the NMS algorithm with Soft-NMS, to observe the impact of these improvements on the model’s performance. The results of the ablation experiments are shown in Table 5.

**Figure 7:**
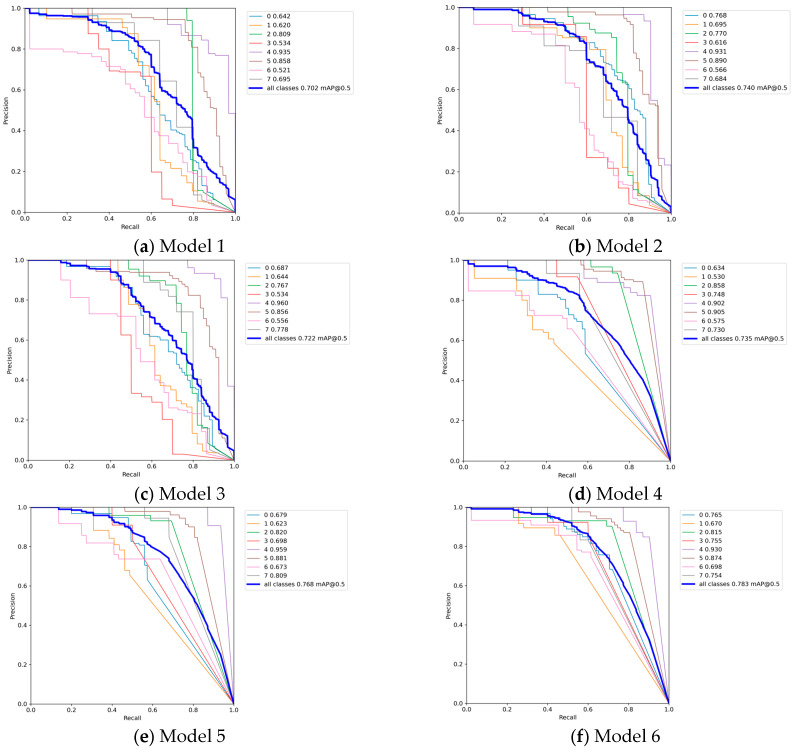
P–R curves of the ablation experiment models.

As displayed in Table 5, the test results using the original SPPF module are not very good. *mAP*@0.5 and *mAP*@0.5:0.95 values are 70.2% and 41.9%, respectively, and the *F*1 score is 69%. After the introduction of the LSKA mechanism, although the *FLOPs* value has risen slightly, *mAP*@0.5, *mAP*@0.5:0.95, and the *F*1 score significantly increased, reaching 74%, 43.2%, and 74%, respectively. The introduction of the DIoU loss function also resulted in a small rise in the *FLOPs* and a relative increase in precision evaluation metrics. After the introduction of Soft-NMS alone, *mAP*@0.5 reached 73.5%, *mAP*@0.5:0.95 reached 46.4%, and the *F*1 score reached 70%. It is worth noting that the introduction of Soft-NMS improved *mAP* more significantly. In summary, the introduction of LSKA into the SPPF module, the replacement of the loss function with DIoU, and the replacement of the NMS algorithm with Soft-NMS all significantly improved the detection accuracy of the models. The P–R curves of each model are shown in Figure 7.

As can be seen in Figure 7, our improved model obtained the highest *AP* values in pothole and strip patch detection, and the second-highest *AP* values in linear crack (longitude and transverse) detection. Our model does not have a strong comparative advantage in the detection of other categories of distress.

Comparing Model 2 with Model 1, the experimental results confirm the superiority of LSKA in processing elongated pavement distress (such as longitudinal and transverse cracks). Conventional 3 × 3 convolutional kernels possess a limited receptive field, making it difficult to capture large-span crack structures. By utilizing large-kernel decomposed convolutions, LSKA establishes long-range spatial dependencies without significantly increasing the parameter count. This enables the model to extract more continuous structural features from complex pavement textures, effectively suppressing background noise interference that mimics crack patterns.

The results from Model 3 indicate that the varying aspect ratios of pavement patches in forward-view images pose a challenge for CIoU. The mandatory aspect ratio consistency penalty in CIoU tends to produce unstable gradient directions when encountering distress with stochastic (random) shapes. In contrast, DIoU directly optimizes the center-point distance between the predicted and ground-truth boxes. This provides more direct geometric constraints when handling such non-rigid targets, resulting in a more stable regression process.

In pavement inspection scenarios, dense distress (such as zebra crossings or alligator cracking) often lead to highly overlapping detection boxes. Traditional NMS employs a “hard deletion” strategy, which frequently misidentifies and removes valid overlapping boxes belonging to distinct distress. Soft-NMS effectively retains these dense targets by decaying their detection scores rather than performing immediate exclusion. This explains why the system’s “error detection and omission prevention” capabilities are significantly enhanced while maintaining high precision, an improvement further validated by the comparison between Model 4 and Model 1.

Ultimately, the experiments demonstrate that the integration of these three modules is not a mere accumulation of components but a synergistic complement across three dimensions: feature extraction (LSKA), regression optimization (DIoU), and post-processing filtering (Soft-NMS). Specifically, LSKA enhances “perception” (the ability to see), DIoU improves “calculation” (the accuracy of localization), and Soft-NMS ensures “comprehensiveness” (the integrity of results). This tailored design allows the improved model to achieve a superior detection balance while preserving the lightweight advantages of the YOLOv8n framework.

**Figure 8:**
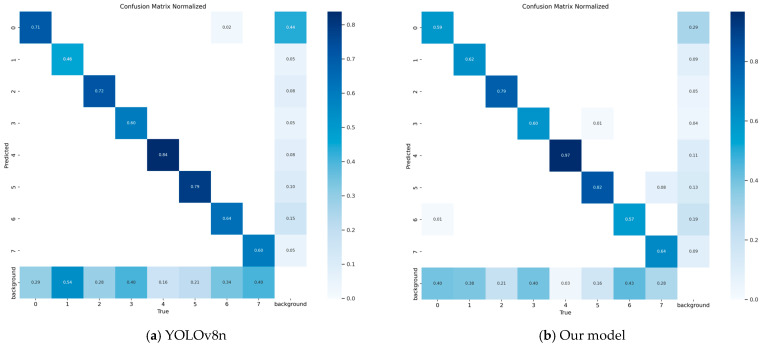
Normalized confusion matrix.

To further investigate the classification performance and identify the specific failure modes of the models, we present the normalized confusion matrices for both the baseline YOLOv8n model and our proposed improved model in Figure 8.

It can be concluded from Figure 8 that the improved model shows remarkable gains in zebra marking (recall of class 4 reaching a near-perfect 0.97) and transverse cracks (a ~35% increase in recall of class 1). This demonstrates that the improvement strategies (LSKA/DIoU) are highly effective for feature extraction in these categories. Recall performance on class 0 (longitude cracks) and class 6 (strip patches) declined slightly. This often suggests a shift in the model’s “discrimination” boundaries.

The bottom row (“background”) represents the probability of the model misclassifying a true target as background. Lower values indicate a lower miss rate (false negatives). The value for transverse cracks (class 1) decreased from 0.54 (baseline) to 0.38 (improved), indicating a significant reduction in missed detections. The value dropped sharply from 0.16 to 0.03, which means that the model is extremely robust, rarely misclassifying zebra marking targets as background, which aligns with the high recall (0.97) mentioned above. Most categories (1, 2, 3, 4, 5, and 7) show a decrease in false negatives. This suggests that the improved model has learned stronger target features, effectively reducing instances where distress is mistakenly identified as pavement.

**Figure 9:**
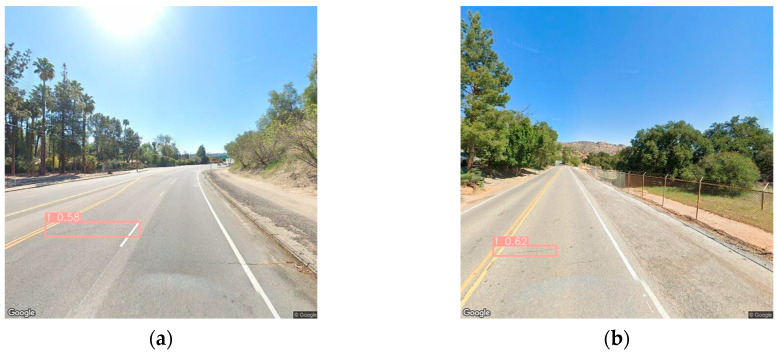
Transverse crack detected results. (**a**) 1st case, (**b**) 2nd case.

**Figure 10:**
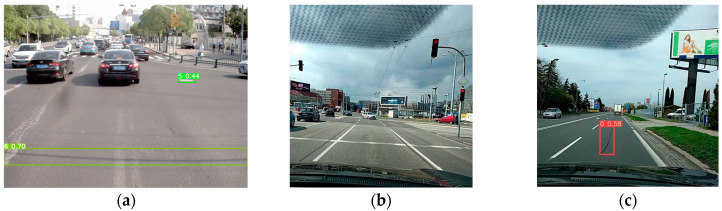
Strip patch results detected by our model. (**a**) 1st case, (**b**) 2nd case, (**c**) 3rd case.

To gain insights into the model’s limitations, we performed a systematic error analysis by selecting representative failure cases. The selected samples are shown in Figure 9 and Figure 10.

As illustrated in Figure 9, several severe transverse cracks on the highway pavement remain undetected. We attribute this failure to the distinct surface characteristics of highway pavements, which typically exhibit lower asphalt content and lighter surface coloration compared to urbanized pavements. Furthermore, the accumulation of surface contaminants within the cracks significantly diminishes their visual contrast against the surrounding road, complicating the feature extraction process. Despite these localized detection challenges, the improved model demonstrates significant superiority over YOLOv8n in capturing linear cracks across more favorable conditions.

Figure 10 showcases typical failure cases in strip patch detection. The model incorrectly identifies a utility pole shadow as a strip patch in Figure 10a, fails to detect the target in Figure 10b, and misclassifies a strip patch as a longitudinal crack in Figure 10c. These errors likely stem from a domain shift between the Shanghai subset and the Czech subset, where the morphological and spectral characteristics of strip patches differ considerably. Notably, shadow-induced false positives represent a persistent challenge. A potential mitigation strategy involves annotating shadow regions (e.g., from utility poles) as auxiliary background context during training to enhance the model’s discriminatory capability. Given the constraints of our current dataset annotations, this task is reserved for future research.

### 4.3. Comparison with Different Model Frameworks

Although the results of the ablation experiments demonstrate the effectiveness of the improved model, we also compared our model with several other state-of-the-art target detection algorithms, including RTDETR-l, YOLOv3-tiny, YOLOv5n, YOLOv6n, YOLOv7-tiny, YOLOv8n, YOLOv8s, YOLOv9c, YOLOv13n, YOLOv26n, and YOLOv8s, and the detailed results of the comparison experiments are shown in Table 5.

According to Table 6, YOLOv3-tiny has the best results in terms of *Latency* and *FPS* but performs poorly in terms of detection accuracy. YOLOv7-tiny and RTDETR-l have lower detection accuracies, and RTDETR-l has the highest model complexity and the longest training time. YOLOv8s has a better detection accuracy, with a *mAP*@0.5 of 0.754 and an *F*1 score of 0.73, but the number of parameters and the value of *FLOPs* of YOLOv8s are high, the model size is larger, and the *FPS* is lower than those of YOLOv8n and the improved model. Our model exhibits a superior balance of detection accuracy and real-time performance on GPU, with its slight latency increase being outweighed by the significant gains in detection precision.

### 4.4. CPU-Based Model Deployment Inference Experiments

The efficiency test results of the CPU-based test experiments are shown in Table 7.

As evidenced in Table 7, the YOLOv5n model with the smallest size is the most efficient, followed by our model. Although YOLOv5n achieves the best performance in the inference computation experiments, its detection accuracy is poorer (as shown in Table 4). Certain models that demonstrate robust real-time performance on GPU, including YOLOv3-tiny, YOLOv6n, and YOLOv8s, exhibit sub-optimal performance on CPU following end-side optimization. This discrepancy can be attributed to several factors. One reason is that when optimized for porting to run on CPU, the complex structure may lead to a significant degradation of real-time performance. In addition, OpenVINO optimizes certain models and operators differently. The models may result in more frequent or irregular memory access on the CPU.

Our model outperforms YOLOv8n in real-time metrics, including *Latency* and *FPS*. The combined accuracy, model complexity, and speed metrics demonstrate that our algorithm strikes a balance between detection accuracy and real-time performance and exhibits superior performance in pavement distress detection tasks.

## 5. Conclusions

This study proposes an enhanced YOLOv8n framework for automated pavement distress detection by integrating the LSKA attention mechanism, DIoU loss function, and Soft-NMS algorithm. Experimental results demonstrate that the proposed method achieves superior detection accuracy while maintaining real-time performance. Key findings are summarized as follows:

(1) The improved model achieves 78.3% mAP@0.5 (+8.1%) and 49.0% mAP@0.5:0.95 (+7.1%) compared to the baseline YOLOv8n, accompanied by a 5% improvement in F1-score. Notably, AP gains of 22.1% for potholes and 17.7% for strip patches validate the model’s enhanced adaptability to small target detection and complex background environments.
(2) Ablation studies quantify the contribution of each module: the LSKA mechanism serves as the primary driver for performance gains (+3.8% mAP@0.5) by suppressing background noise; Soft-NMS significantly reduces false negatives (+3.3% mAP@0.5); and the DIoU loss optimizes bounding box regression accuracy.
(3) While the model experiences marginal increases in computational complexity (FLOPs: 8.3 G, +0.2 G) and parameter size (6.8 M, +0.5 M), it sustains high real-time efficiency, maintaining 160 FPS on GPU and 68 FPS on CPU. This confirms that the proposed model achieves a superior balance between detection precision and computational cost compared to the baseline.
(4) Extensive benchmarking against state-of-the-art models—including YOLOv3-tiny, YOLOv5n, YOLOv6n, YOLOv7-tiny, YOLOv8n, YOLOv8s, YOLOv9-c, YOLOv13n, YOLOv26n, and RTDETR-l—confirms the method’s effectiveness in balancing accuracy and speed.

While our use of a multi-source dataset has enabled the model to learn robust, domain-invariant features, future work will focus on large-scale validation across geographically diverse regions and varying meteorological conditions. This will further benchmark the upper bounds of the model’s generalizability and substantiate its reliability for large-scale, real-world infrastructure deployment.

## Figures and Tables

**Table 1:** Comparison of attention mechanisms.

Mechanism	Focus	Receptive Field	Complexity	Suitability for YOLOv8n
SE [35]	Channel	Global (Pool)	Low	Limited (ignores spatial structure)
CBAM [36]	Channel + Spatial	Local (7 × 7)	Moderate	Sub-optimal for elongated cracks
Transformer [37]	Spatial	Global (MHSA)	High (O(N^2^))	Too heavy for real-time edge devices
LSKA (Ours)	Spatial	Global	Low (Linear)	Optimal (Efficiency + Receptive Field)

**Table 2:** Comparison results based on the test set (5 runs).

Model	mAP@0.5 (%)	mAP@0.5:0.95 (%)	F1 (%)	Params (M)	FLOPs (G)	Latency (ms)	FPS	Model Size (M)
YOLOv8n	68.7 ± 1.5	40.8 ± 1.1	67.7 ± 1.3	3.007	8.1	6.15 ±0.31	162.6	6.3
Ours (Improved YOLOv8n)	76.7 ± 1.3	48.3 ± 0.7	72.9 ± 1.1	3.28	8.3	6.24 ± 0.11	160.3	6.8

**Table 3:** Optimal model’s detected AP results for different categories based on the test set.

Model	*AP*@0.5 (%)							
	Longitude Cracks	Transverse Cracks	Net Crack	Pothole	Zebra Crossing Loss	Manhole	STRIP Patch	Block Patch
YOLOv8n	64.2	62	80.9	53.4	93.5	85.8	52.1	69.5
Ours	76.5	67	81.5	75.5	93	87.4	69.8	75.4
Increase	+12.3	+5	+0.6	+22.1	−0.5	+1.6	+17.7	+5.9

**Table 4:** AP results for different categories based on the SCVRDD dataset.

Distress type	Images	Instances	YOLOv8 AP50	YOLOv8 mAP50–95	Ours AP50	Ours AP50–95
longitudinal_crack	1000	567	0.451	0.248	0.471	0.259
transverse_crack	1000	473	0.462	0.238	0.476	0.254
alligator_crack	1000	260	0.508	0.290	0.545	0.301
pothole	1000	131	0.387	0.126	0.362	0.161
manhole_cover	1000	452	0.666	0.379	0.672	0.388
longitudinal_patch	1000	961	0.600	0.366	0.593	0.351
transverse_patch	1000	406	0.471	0.237	0.484	0.245
Average (Mean)	-	-	0.506	0.269	0.515	0.280

**Table 5:** Ablation test results. Note: √ indicates that the module is used, while × indicates that it is not used.

Model No.	Modules			mAP@0.5 (%)	mAP@0.5:0.95 (%)	F1 (%)	FLOPs (G)
	LSKA	DIoU	Soft-NMS				
1	×	×	×	70.2	41.9	69	8.1
2	√	×	×	74	43.2	74	8.3
3	×	√	×	72.2	42	70	8.1
4	×	×	√	73.5	46.4	70	8.1
5	×	√	√	76.8	47.9	72	8.1
6 (Our model)	√	√	√	78.3	49	74	8.3

**Table 6:** Comparative results of different models. Note: bold indicates the optimal value.

Model	*mAP*@0.5 (%)	*mAP*@0.5:0.95 (%)	*F*1 (%)	*Params* (M)	*FLOPs* (G)	*Latency* (ms)	*FPS*	*TT* (h)	*Model Size* (M)
RTDETR-l	62.7	35.6	60	32	103.5	19.07 ± 0.47	52.4	2.425	66.2
YOLOv3-tiny	63.2	33.1	62	12.13	18.9	2.63 ± 0.04	379.8	0.421	24.4
YOLOv5n	71.4	41.2	72	2.50	7.1	6.87 ± 0.07	145.5	0.446	5.3
YOLOv6n	66	37.6	63	4.23	11.8	6.02 ± 0.12	166.1	0.374	8.7
YOLOv7-tiny	64	35.7	62	6.03	13.1	6.60 ± 0.39	151.4	0.772	12.3
YOLOv8n	70.2	41.9	69	3.01	8.1	6.15 ± 0.31	162.6	0.396	6.3
YOLOv13n	57.5	30.4	57	2.83	6.4	/	/	/	5.7
YOLOv26n	69.2	43.8	70	**2.38**	**5.2**	/	/	/	**4.8**
YOLOv8s	75.4	45.6	73	11.13	28.5	6.91 ± 0.13	144.7	0.547	22.5
YOLOv9-c	76.3	48.7	**75**	50.71	236.7	28.72 ± 0.67	34.8	/	98.1
**Ours**	**78.3**	**49**	74	3.28	8.3	6.24 ± 0.11	160.3	0.423	6.8

**Table 7:** Comparative efficiency results of the CPU-based reasoning experiments.

Model	*Latency* (ms)				*FPS*	*Model Size* (M)
	Median	Average	Min	Max		
YOLOv3-tiny	5601.65	7040.15	900.34	32,692.22	17.95	46.3
YOLOv5n	497.55	891.18	14.02	9495.56	71.49	9.7
YOLOv6n	507.85	1894.55	115.76	9597.07	33.66	16.3
YOLOv8n	596.57	989.59	200.72	7291.52	64.4	11.7
YOLOv8s	504.96	1520.58	105.48	18,200.6	41.9	42.6
Our model	506.57	934.76	114	8701.4	68.07	12.7

## Data Availability

The data presented in this study are available on request from the corresponding author due to the restriction of the company [China Merchants Expressway Network & Technology Holdings Co., Ltd.].
